# Robust In-Plane Structures Oscillation Monitoring by Terrestrial Photogrammetry

**DOI:** 10.3390/s20082223

**Published:** 2020-04-15

**Authors:** Omar El-Kadi, Adel El-Shazly, Khaled Nassar

**Affiliations:** 1 Civil Engineering, Faculty of Engineering, Cairo University, Giza Governorate 12613, Egypt; Adel_shazly@hotmail.com; 2 Construction Engineering Department, The American University in Cairo, Cairo 11865, Egypt; knassar@aucegypt.edu

**Keywords:** automation, deep learning, deformation monitoring, Faster R-CNN, image processing, oscillation monitoring, non-metric camera, terrestrial photogrammetry, tensor flow, video processing

## Abstract

Oscillation monitoring commonly requires complex setups integrating various types of sensors associated with intensive computations to achieve an adequate rate of observations and accuracy. This research presents a simple, cost-effective approach that allows two-dimensional oscillation monitoring by terrestrial photogrammetry using non-metric cameras. Tedious camera calibration procedures are eliminated by using a grid target that allows geometric correction to be performed to the frame’s region of interest at which oscillations are monitored. Region-based convolutional neural networks (Faster R-CNN) techniques are adopted to minimize the light exposure limitations, commonly constraining applications of terrestrial photogrammetry. The proposed monitoring procedure is tested at outdoor conditions to check its reliability and accuracy and examining the effect of using Faster R-CNN on monitoring results. The proposed artificial intelligence (AI) aided oscillation monitoring allowed sub-millimeter accuracy monitoring with observation rates up to 60 frames per second and gained the benefit of high optical zoom offered by market available bridge cameras to monitor oscillation of targets 100 m apart with high accuracy.

## 1. Introduction

Deformation monitoring is an essential task in the field of geomatics, with vast fields on applications as landside deformations monitoring, monitoring of slopes and rock stability, structures and bridges deformation monitoring, and many more. Modern technologies and sensors used with digital photogrammetry, allow the usage of photogrammetry in deformation monitoring, and facilitate the implementations of new techniques and approaches in monitoring procedure. Unmanned Aerial Vehicles (UMAV) used in aerial photogrammetry is often defined as a drone, the resolution of the drone’s detection depends on the altitude and the characteristics of the camera [1]. Modern quadcopters can achieve a spatial resolution of one to three centimeters [2], which can be used for landslide deformation monitoring [3]. Moreover, different systems of monitoring using close-range photogrammetry were tested [4], some systems are adopted for commercial use as dynamic monitoring station system, which was commercialized by university of Bristol, at the United Kingdom, in 2003 [5], the use of off-shelf modern digital cameras became a concern, and was tested for monitoring application by many researchers achieving sub-millimeter precision for both static and dynamic deformations [6,7].

The use of non-metric cameras in photogrammetry may be sufficiently accurate if narrow cone angles and analytical methods are employed [8]. However, yet many standards limit deformation monitoring applications by photogrammetry to the use of a metric camera [9,10]. Another limitation of applying photogrammetry for deformation monitoring is the effect of illumination variation due to the change of lighting conditions and shadows from surrounding objects in the monitoring environment, which can lead to failure of target recognition in several successive images or specific monitoring periods [5]. The modern available computing powers, associated with rapid development in neural networks techniques and deployment, add extra capabilities to the implementations of digital photogrammetry in different monitoring applications by felicitating object detection, classification, and segmentation tasks. Many of the used algorithms are based, or developed from the concept of convolutional neural networks (CNNs) that features local receptive fields, shared weights and biases, and activation and pooling. As a result, defect detection and cracks segmentation in engineering structures [11,12] can be achieved by digital photogrammetry featuring masked region proposals convolutional neural networks (Mask R-CNN) networks [13]. Nevertheless, monitoring by photogrammetry can theoretically be in real-time, but the computational processes and powers required for analysis commonly limit the monitoring to be near real-time. As an example, the polynomial and the rational models used for image rectification cannot be adapted to global bundle adjustment, where the internal/external parameters and the distortion model are estimated simultaneously [14].

The development of approaches implemented for deformation and oscillation monitoring is going on in rapid cycles, as new techniques for using synthetic aperture radar are applied for deformation monitoring. The usage of ground base differential interferometry synthetic aperture radar promises significant improvement in continuous monitoring of steep slopes and embankments [15] and overcomes many of space-borne interferometric synthetic-aperture radar (InSAR) limitations [16].

## 2. Used Sensor and Models

### 2.1. Monitoring Camera

**Figure 1:**
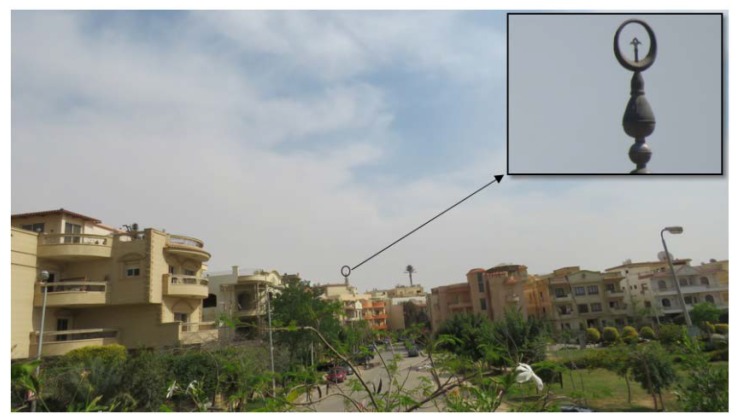
Example of 65× optical magnification power obtained using Canon Powershot Sx60hs camera for object about 330 m away.

Bridge cameras offer a wide range of superzoom and resolution with a relatively low price compared to professional cameras, Nikon Coolpix P900 offers 83× zoom with 16 MP still image resolution, and video with a 1920 × 1080-pixel resolution at 60 frames per second, while Nikon Coolpix P1000 offers 125× zoom, Table 1 presents most commonly market available cameras specifications, while the effectiveness of optical zoom is presented in Figure 1 as a target 330 m apart is pictured using 65× optical zoom by Canon Powershot SX60Hs camera.

#### Camera’s Sensor Precision Analysis

The resulting monitoring accuracy is a function of the camera’s pixel precision. Accordingly, pre-analysis is performed on the used camera (Canon Powershot sx60hs); and Nikon Coolpix P1000, which is a widely available bridge camera. The analysis is based on the assumption of monitoring target from 100 m away stations, and applying the relation of digital image scale, as the by-product of camera focal length and the field of view equal to the by-product of sensor’s size and monitoring distance. Meanwhile, pixel precision equals the division of minimum Field of view (FoV) by image’s number of pixels on monitoring used camera as:

(1) Focal length*FoV=sensor size*monitoring distance

(2) $$Pixel\;precision=\frac{Minimum\;FoV}{Number\;of\;pixels}$$

Canon Powershot SX60HS has a CMOS sensor with a size of 1/2.3″ (6.17 mm × 4.55 mm) that can capture 6.4 images per second with a resolution of 4608 × 3456 pixels or video of 1920 × 1080 at 60 fps and has a focal length range of 3.8–247.0 mm. Accordingly, for a target 100 m apart monitoring precisions of 1.3 mm and 1.7 mm in the x and y direction, respectively, are expected with video monitoring at a resolution of 1920 × 1080; While monitoring precisions of 0.54 and 0.533 mm in x and y direction, respectively, are expected with continuous still images monitoring (limited to six images per second), Figure 1, shows an example as 65× optical zoom is used to target an object 330 mm apart, resulting in a pixel pitch of 1.78 and 1.76 mm in x and y directions when using a resolution of 4608 × 3456 pixels. On the other hand, Nikon Coolpix P1000 camera is powered with a medium-size CMOS sensor of size 1/2.3″ (6.17 mm × 4.55 mm) and able to capture seven images per second with a resolution of 4608 × 3456 pixels or video of 1920 × 1080 with 60 fps, and has a focal length range of 4.3–539 mm, reaching a zoom of 125×. Accordingly, for a target 100 m apart monitoring precision of 0.596 and 0.782 mm in x and y direction is expected with video monitoring at a resolution of 1920 × 1080; And monitoring precisions of 0.248 and 0.244 mm in x and y direction, respectively, are expected with continuous still images monitoring (limited to seven images per second), a summary of camera sensors precision for monitoring target 100 m apart is presented Table 2.

### 2.2. Effect of Refraction and Out of Plan Movements on Two-Dimensional Monitoring

Close range photogrammetry is commonly used with target distance less than 100 m. In this case, the effect of a line of sight refraction due to atmospheric temperature can be ignored. However, the superzoom capabilities offered by modern bridge cameras allow larger target distance (as presented in Figure 1). The proposed monitoring method is based on two-dimensional relative drifts, at which the effect of refraction is assumed to be eliminated by differencing during deformations calculations.

**Figure 2:**
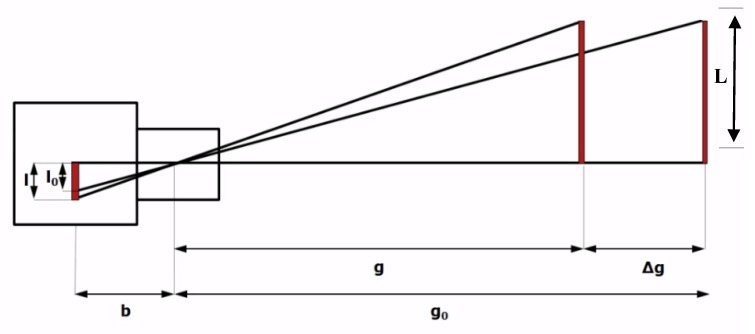
Effect of out of plan movement on monitoring 2D deformations [20].

Figure 2 illustrates the resulting error (*ε*), produced by the out of plane movement of the target (Δ*g*), that will result in a new projection of object image *I* instead of *I_o_*. the resulting error can be computed as:

(3) $$\varepsilon =I_{o}-I=\left(\frac{L}{g_{o}}-\frac{L}{\left(g_{o}-\Delta g\right)}\right)xb$$

L_max_ = FoV/2 (4)

By applying previous equations for Canon Powershot sx60hs camera monitoring target 100 m with maximum optical zoom of 65×, the focal length of 247 mm is be used, resulting in minimum FoV of 2497.975 mm in the X-direction, and minimum FoV of 1842.10 mm in the Y-direction, when capturing video at a resolution of 1920 × 1080 pixels. Assuming inward target movement of 1 m, error in different directions can be calculated from Equation (3) as ε_Xmax_ = −0.033 mm, and ε_ymax_ = −0.023 mm. While for a target of 500 mm × 500 mm dimensions monitored five meters apart, the focal length of 45.5 mm is required to have sufficient FoV, resulting ε_Xmax_ = −0.063 mm, and ε_ymax_ = −0.046 mm, at image frame boundary, due to 10 cm inward motion of the target.

From a previous analysis, it can be concluded that, out of plane movements of 1 m and 10 cm, when monitoring from stations 100 m and 5 m, results in maximum error less than 0.1 mm at the image boundary, and zero error is expected at the image center. Accordingly, the effect of out of plan movement can be ignored.

### 2.3. Images Geometric Corrections and Removal of Lenses Distortion

To use non-metric bridge cameras for oscillation and deformation monitoring images, geometric errors, and distortion of lenses should be considered. Commonly, an in-situ calibration process is performed prior to the observations procedure, at which the camera geometric model is calculated together with distortion parameters, whereas in applying Zhang’s camera calibration procedure [21]. While, if control points used for calibration are not coplanar, direct linear transform may be applied.

The introduced method does not require camera calibration but benefits from the case that three-dimensional reconstructions are not desired and implements coplanar control points along a grid to determine coefficients of distortion function. Using a target similar to that used in Zhang’s calibration procedure [22], during the monitoring procedure. 

#### 2.3.1. Image Geometric Correction Based on Nonlinear Distortion Model

Camera lenses have mostly radial distortion and little tangential distortion; radial distortions can be calculated by using an odd powered polynomial series [23]. Moreover, open-source libraries as OpenCV facilitate the computation of radial distortion coefficients (k_1_, k_2_, and k_3_) as:

(5) $$r^{2}={\left(x_{i}^{u}\right)}^{2}+{\left(y_{i}^{u}\right)}^{2}$$

(6) $$X_{i}^{c}={\;x}_{i}^{u}\;\left(1+k_{1}r^{2}+k_{2}r^{4}+k_{3}r^{6}\right)$$

(7) $$Y_{i}^{c}={\;y}_{i}^{u}\;\left(1+k_{1}r^{2}+k_{2}r^{4}+k_{3}r^{6}\right)$$

And tangential distortion coefficients (p_1_ and p_2_) as:

(8) $$X_{i}^{c}={\;x}_{i}^{u}+\left(2\rho_{1}\mathrm{xy}+\rho_{2}\left(r^{2}+2x^{2}\right)\right)$$

(9) $$Y_{i}^{c}={\;y}_{i}^{u}+\left(2\rho_{2}\mathrm{xy}+\rho_{1}\left(r^{2}+2x^{2}\right)\right)$$

where:

$x_{i}^{u}\;and\;y_{i}^{u}:$ are uncorrected pixel’s coordinates
$X_{i}^{c}\;and\;Y_{i}^{c}:$ are corrected pixel’s coordinates

The target points are detected by a Harris corner detector [24], with subpixel accuracy based on gradient direction, and neighborhood search [25]. That allows the distortion parameters to be computed by Equations (5)–(9) based on the relation between detected gridded target points, and their predefined correct locations. Then, those parameters are used to project the image to have an undistorted image.

This research proposes a technique to avoid an undesired calibration process before the monitoring procedure by using a target of a uniform grid for the monitoring procedure. This target’s initial observation is used to estimate the distortion coefficients as stated above and obtain undistorted images for the target for different monitored images. Followed by projective transform estimated form initial undistorted image to ensure minimal geometric errors. The parameters estimated from the projective transform are used for geometric corrections for successive observations, resulting undistorted region of interests at targets area, at which maximum oscillation drift is assumed to be smaller than half the target width. 

#### 2.3.2. AI Aided Target Detection by Faster R-CNN Network

**Figure 3:**
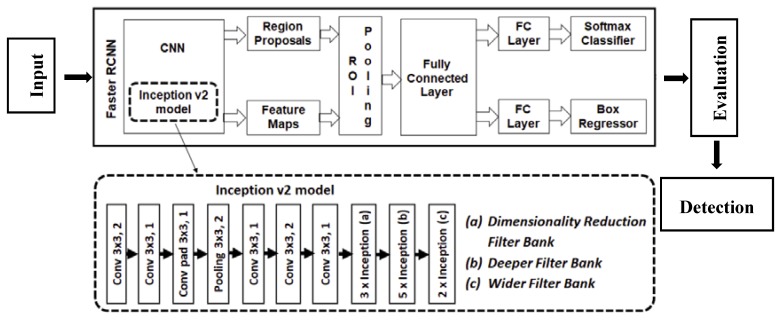
Structure of Faster RNN network with inception V2 model [28].

Despite using radiometric corrections as histogram equalization and histogram matching techniques, the variation of light exposure turns target detection into a challenge facing deformation and oscillation monitoring by photogrammetry, especially at outdoors applications like structural monitoring, as presented in the case of Humber and Tamar bridges monitoring using close-range photogrammetry, [5]. On the other hand, the rapid development in neural networks techniques and deployment allows object detection in various light exposure conditions, based on a deep learning process that trains the network to detect targets in such situations. Faster R-CNN is a developed algorithm that is based on the approach of region-based convolutional networks, while a discrete network that is used to forecast the region proposals [26]. Faster R-CNN with inception V2 model presented in Figure 3 is a fast and efficient algorithm that constructs an inception v2 network from inputs to the given final endpoint up to the layer inception (5b) as described in [27]. This network has been implemented to detect two classes, which are the target and a predefined marked tracking point (named Track-point).

**Figure 4:**
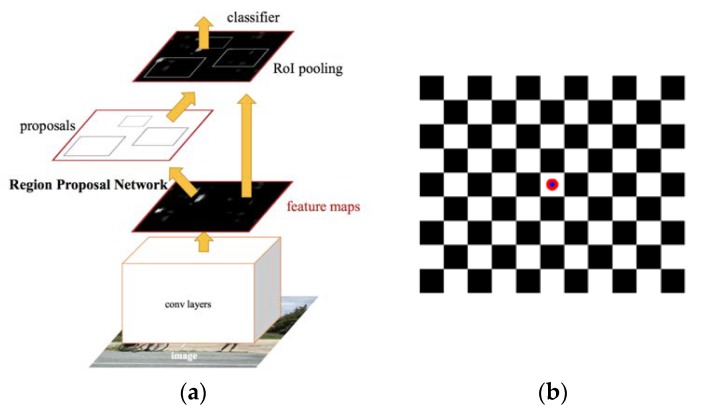
(**a**) Architecture of Faster Region-based convolutional neural networks (R-CNN) [29], (**b**) Target with Track-point [30].

The Track-point, as presented in Figure 4b, is selected to have a circular shape in order to reduce classifier box shifting at various image shooting angles. The Track-point was defined with a pattern of red and blue colors to have a distinctive light intensity pattern that minimizes false detection of surrounding features.

The artificial intelligence (AI) aided tracking is assumed to be robust in various lighting conditions. However, this proposed technique is assumed to have less spatial precision, compared to Harris corner detector applying gradient direction, and neighborhood search for subpixel accuracy used earlier in Section 2.3.1.

**Figure 5:**
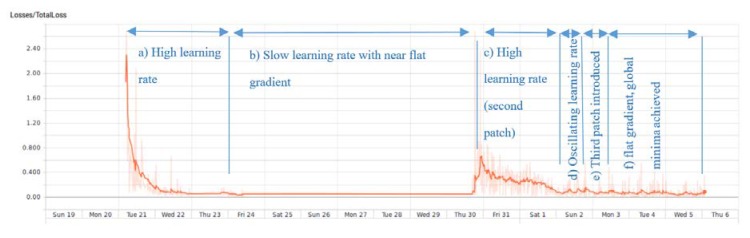
Loss function vs. time during network training until learning step 28330.

Tensor Flow application programming interface (API) [31] has been used to train the network, applying a transfer learning form model pre-trained [32] on the Common objects in context (COCO) dataset [33]. The training was performed over three phases to check the training performance. In the first phase, the network was trained by about 300 labeled images for the learning dataset and 40 labeled images for the testing dataset. Then learning is applied to the second and third learning stages, using about 150 new images per phase for the learning dataset and 40 images for the testing dataset. The network training was performed based on CPU processor computations due to the usage of relatively large images in the training dataset (about 600 × 1000 pixels), resulting in GPU memory overflow even when reducing training patches to one image. As a result, the training process consumed about eight days for training for the first phase, and four days per each successive phase. The loss function was used to indicate network training, as presented in Figure 5. Images with the resolution of 8 MP, 12 MP, 16 MP, and 1920 × 1080 pixels were used as input to maintain the grade of detail in the images compared to images obtained by monitoring.

Network evaluation is performed applying mean average precision (mAP) open source code [34], by using 138 images, including 139 Targets and 138 Track-points. Average precision (AP), mean average precision (mAP), recall, and intersection over union (IoU) [35,36] are used for the network’s performance evaluation, where IoU refers to the degree of coincidence between the detected area and the ground truth area as:

(10) $$\mathrm{IoU}=\frac{\mathrm{Area}\;\left(B_{p}\cap B_{\mathrm{gt}}\right)}{\mathrm{Area}\;\left(B_{p}\cup B_{\mathrm{gt}}\right)}$$

**Figure 6:**
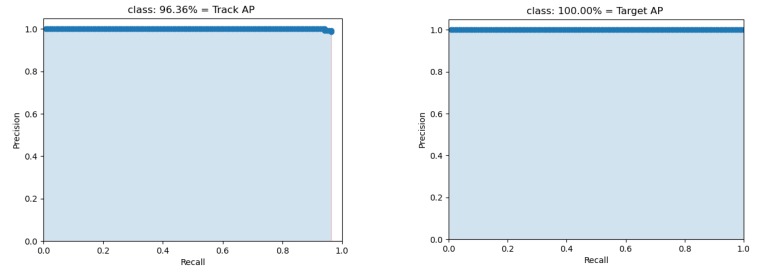
Precision–Recall curves for detected classes.

In Equation (10), Bp and Bgt are the predicted and ground truth bounding boxes. The detection is considered as a true positive (TP) if IoU exceeds 0.5, while precision and recall are calculated as follows:

(11) $$\mathrm{Precision}=\frac{\mathrm{TP}}{\mathrm{FP}+\mathrm{TP}}$$

(12) $$\mathrm{Recall}=\frac{\mathrm{TP}}{\mathrm{FN}+\mathrm{TP}}$$

where FP is false positive, and FN is a false negative. The average precision (AP) is calculated by obtaining the area under the precision-recall curve, and the mean average precision (mAP) is calculated by obtaining the mean of calculated AP. The calculated mAP of evaluation data is 98.14%, while evaluation metrics are presented in Figure 6 and Table 3. 

**Figure A1:**
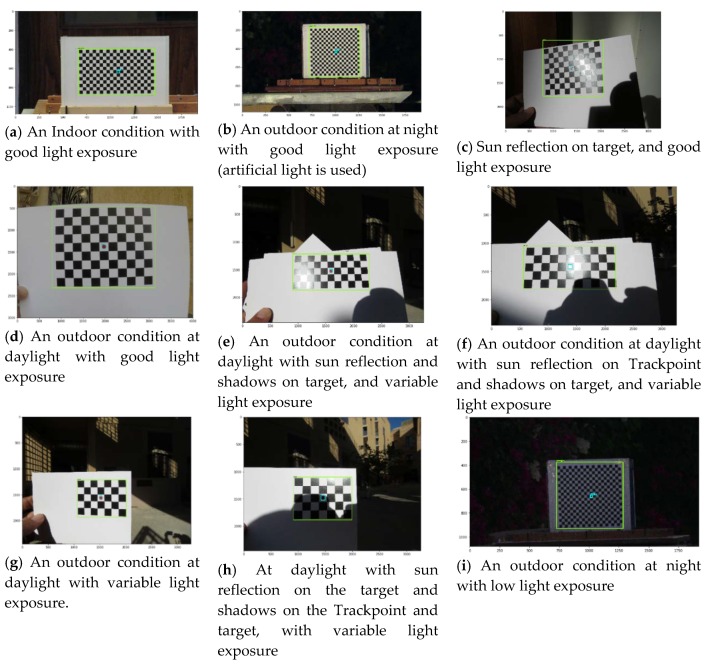
Example of detecting different target classes using trained Faster R-CNN.

As presented in Appendix A, Figure A1, the trained network was able to detect both classes Target and Track-point at different lighting conditions and from different shooting angles. The network was able to detect the target and Track-point when covered with shadows or direct sun reflections. 

#### 2.3.3. Monitoring Work Flow

This research proposes a technique to avoid an undesired calibration process prior to monitoring procedure by using a target of a uniform grid for the monitoring procedure. This target’s point’s observations are used to estimate the distortion coefficients based on different selected frames decomposed from monitoring video, while parameters projective transform are computed from the target’s initial observation, then they are used for geometric corrections for successive observations. This proposed technique results in an undistorted region of interests at targets area, at which maximum oscillation drift is assumed to be smaller than half the target width. 

**Figure 7:**
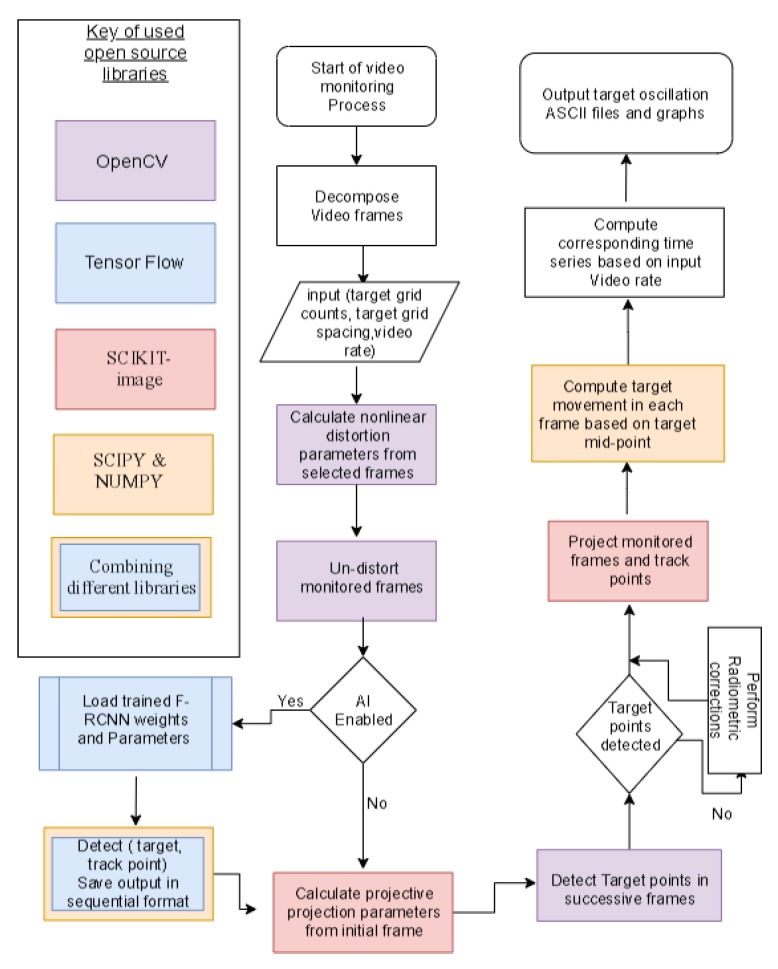
Flowchart presenting proposed monitoring workflow.

The workflow presented in Figure 7 is automated via a python script. The script implements open source libraries NUMPY [37], SCIPY [38,39], SCIKIT—image libraries [40], and OpenCV [41]; That facilitates matrix operations, computational operations, and application of various digital image processing functions, and allowed the automation of monitoring procedures via a developed program implementing different functions form those libraries.

## 3. Examining Consistency and Precision of the Proposed Monitoring Technique

### 3.1. Checking Consistency of Proposed Monitoring Technique

The consistency of the proposed monitoring technique is examined by a test that was conducted in the structural laboratory at the American University in Cairo. Indoor conditions with controlled light exposure is maintained. The purpose of this set of tests is to examine the consistency of the proposed photogrammetric approach compared to linear variable differential transformer (LVDT) measurements, focusing on the ability to match measured by different systems in order to evaluate the precision of oscillation monitoring by terrestrial photogrammetry compared to LVDT.

**Figure 8:**
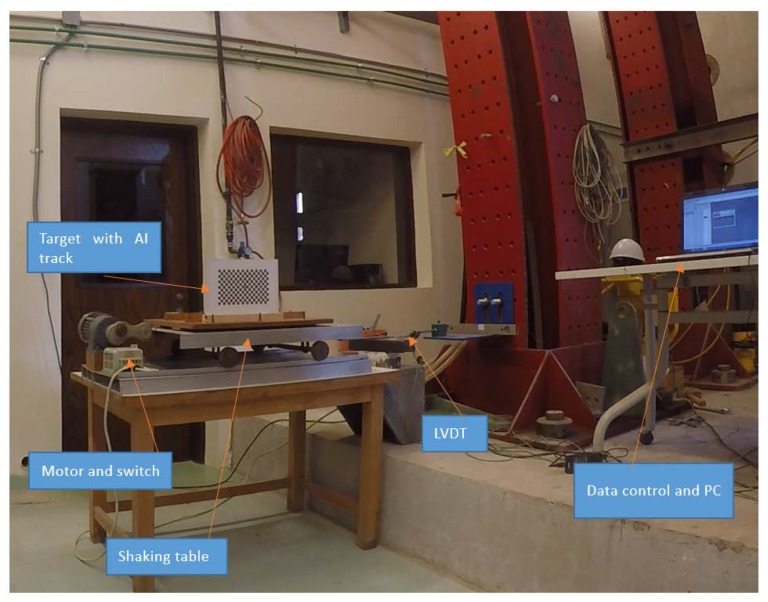
Monitoring of shaking table oscillations test setup.

As shown in Figure 8, a target with a 21 × 12 grid is attached to a small shaking table. The shaking table is powered by an electric motor allowing it to oscillate. The table is rested over four guided metal wheels that control the movement of the table, while the oscillation is measured using LVDT that is connected to the computer via a data logger. The data logger transforms the change in LVDT voltage reading into linear measurement, while a power supply is used for LVDT 10-V excitation.

The monitoring station was placed at about 3.5 m from the target, and the monitoring is performed by a video of 1920 × 1080 pixels at 60 fps and has a field of view covering about 50 cm in the X-direction which results in theoretical monitoring accuracy of 0.26 mm. The used LVDT has a nonlinearity factor equals to 0.4% of the LVDT full scale resulting precision of 0.4 mm, and data controller was set to have data acquisition rate of 50 Hz. 

**Figure 9:**
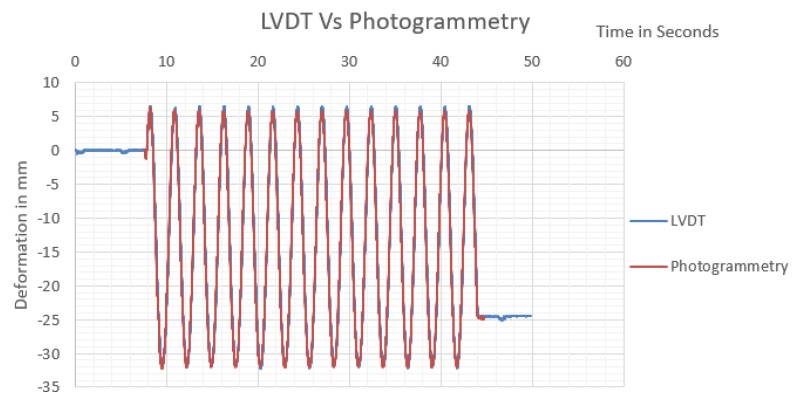
Linear variable differential transformer (LVDT) measured oscillation results vs. photogrammetry monitoring using a Harris corner detector.

**Figure 10:**
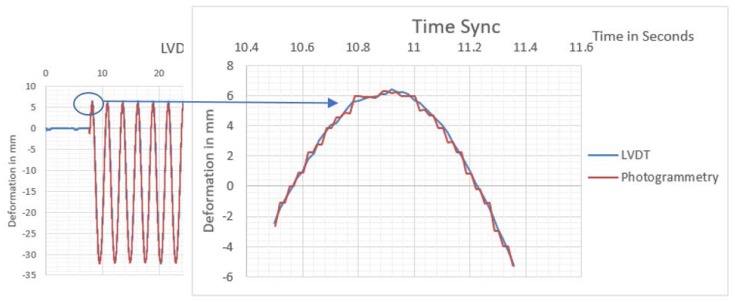
Time-domain synchronization between LVDT and photogrammetry measurements.

The monitoring observations nearly coincided with LVDT measurements, as presented in Figure 9. Both AI and ordinary target tracking monitored waves nearly identically matched. The monitoring video properties indicated that the video had a capturing frequency of 59.94 fps, and the first peak in monitored results was used to synchronize the time domain between LVDT and photogrammetry monitoring, as presented in Figure 10.

The table was oscillating with a frequency of about 0.36 Hz, and a drift of about 38 mm, to have a speed of about 13.68 mm/s. As the comparison between video monitoring and LVDT monitoring is made based on differencing of observations at the nearest timing, 0.05 mm error is expected due to the difference in data acquisition rates of both systems. Accordingly, the maximum error of 0.71 mm is expected theoretically between LVDT and monitoring system, resulting from the LVDT nonlinearity factor, video monitoring precision, and difference of data acquisition rates. While other factors as the efficiency of data logger grounding and real LVDT accuracy can affect results. The maximum and minimum error measured between the LVDT and monitoring system, and the root mean square error of observations are presented in Table 4.

It can be concluded from the analysis results, that both proposed target tracking techniques and geometric corrections allowed successful target tracking with good precision, while the accuracy of analysis cannot be concluded as the reliability of using one LVDT is not high enough to consider it as reference measurements. Moreover, the target of applying AI tracking is to allow monitoring in various light exposure conditions, where a Harris corner detector may fail to detect target grid points. Accordingly, another set of tests are conducted to evaluate monitoring accuracy and reliability in various lighting conditions.

### 3.2. Examining the Precision of the Proposed Monitoring Technique

To check the precision of the proposed monitoring technique, a set of tests was conducted at the American University in Cairo, in an outdoor condition. Three LVDTs were attached to a shaking table, and two monitoring stations were used. The first monitoring station is 4 m apart from the target, while the second station was 28 m apart from the target.

**Figure 11:**
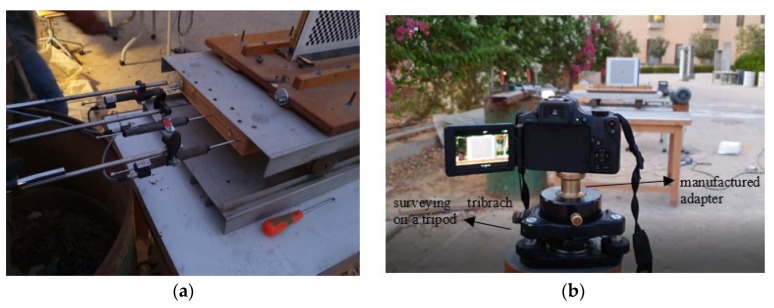
(**a**) Three LVDTs attached to the shaking table and (**b**) monitoring the shaking table from the first station.

As presented in Figure 11b, the camera was mounted on a surveying tripod used a specially manufactured adapter that allowed using surveying tribrach, in order to ensure stability at a windy outdoor condition. Three LVDT’s props were glued to the table, as shown in Figure 11a, to avoid LVDT spring, late response relative to the table oscillation speed. The readings of the three LVDTs are measured at each time instant, with a logging rate of 10 ms (100 Hz), and the average and standard deviation of readings are calculated, the reading with error more than three times the standard deviation is neglected from the average readings of LVDTs. For the oscillation monitoring using terrestrial photogrammetry, a video of 60 fps was used with a resolution of 1920 × 1080, while the captured video metadata showed that the video had a framerate of 59.94 fps which is used later in the analysis.

**Figure 12:**
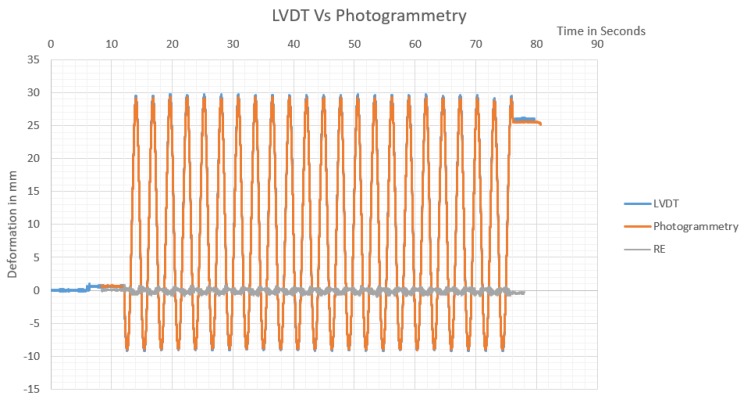
Shaking table monitored oscillations using LVDTs vs. photogrammetry from the first monitoring station.

In the first setup using monitoring station at four meters apart from the target, the shaking table had an average speed of 29.3 mm/s, while the maximum error found between LVDT reading before corrections were 4.26 mm, the high errors were found resulting from the same LVDT which indicated that it has a malfunction. After removing bundle errors more significant than three-times the standard deviation, the maximum relative error in LVDT reading was found to be 0.218 mm, with an RMSE of 0.06 mm. The resulting monitoring by the LVDT system versus monitoring by photogrammetry is presented in Figure 12.

The data acquisition system logging LVDTs readings was affected by noise resulting from lack of good earthling for data logger, as ten voltages are used as exiting current for the LVDT. As shown in Figure 12, nearly after 5 s of monitoring, the average readings of LVDTs deviated from balanced zero reading to be 0.5925 mm, with a maximum error of ±0.787 mm, while the shaking table has not started oscillating yet. Accordingly, during signals matching that is performed for the sake of precision analysis, a drift of 0.5925 mm was added as the initial position, resulting in an expected added error between LVDT and photogrammetry of ±0.1945 mm. The maximum noise in LVDT at the end of monitoring was found to be ±0.4763 mm. The average drift in LVDTs end position was found to be 0.31 mm compared to the photogrammetry end position.

Manual signal matching was used to compare observations obtained by photogrammetry, to that obtained by the LVDTs system, the observations were matched based on the nearest time with a maximum time delay of 0.005 s due to different monitoring rates, resulting in an average error of ±0.146 mm considering the average speed of shaking table. Accordingly, the precision of LVDT monitoring system (considered as reference system) can be considered as ±0.4 mm due to LVDT nonlinearity factor, in addition to ±0.146 mm from time matching, and ±0.4763 mm from LVDT excitement noise, resulting in the precision of ±1.0223 mm. The maximum difference between observations measured using LVDTs system and photogrammetry was found to be 0.8902 mm, and the minimum difference was –1.020 mm, resulting in a maximum residual error (RE) of ±1.020 mm, and root means square error (RMSE) of ±0.351 mm, as shown in Figure 12.

**Figure 13:**
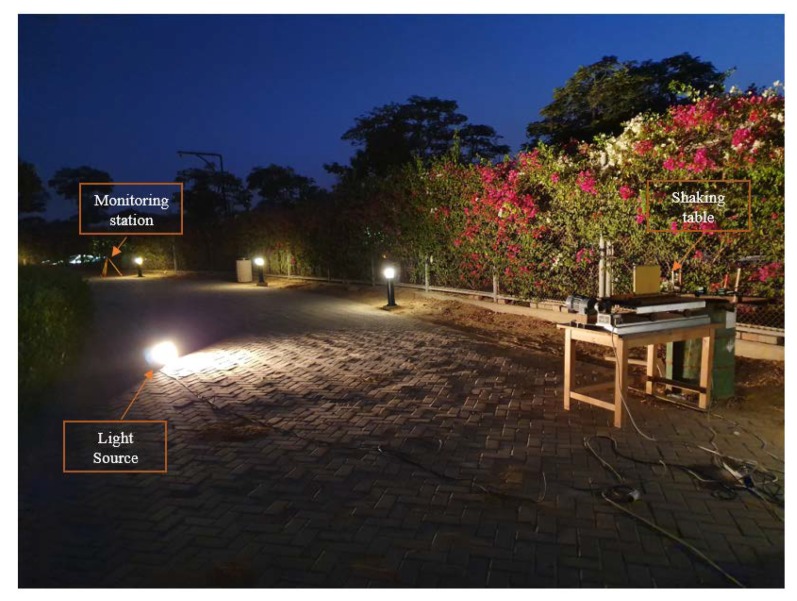
Monitoring shaking table from the second station (28 m apart), at night condition.

Monitoring from the second station was conducted at night using a direct artificial lighting source to examine the ability of the proposed monitoring system in different light exposures, as shown in Figure 13. Moreover, the station is set 28 m apart from target to simulate different required site conditions for monitoring. The field of view at adjusted camera focus was found to be 807.715 mm and 451.334 mm in the x and y directions, respectively. Accordingly, for monitoring using a video of 1920 × 1080 pixels’ accuracy of 0.421 and 0.418 mm is expected in the x and y directions, respectively.

**Figure 14:**
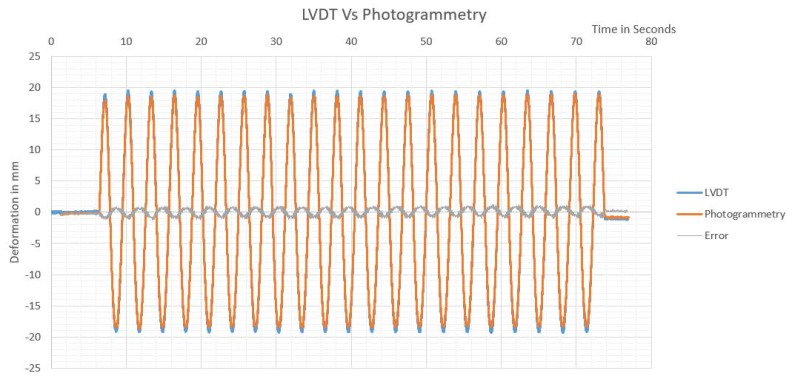
Shaking table monitored oscillations using LVDTs vs. photogrammetry from the second station.

As shown in Figure 14, at the start of monitoring, the average readings of LVDTs did not deviate from balanced zero as happened in the first station, a maximum error of ±0.235 mm was calculated between LVDTs before the shaking table started oscillating. Furthermore, the maximum noise in LVDT at the end of monitoring was found to be ±0.4142 mm.

The expected precision of LVDT monitoring system is ±1.2167 mm, resulting from time matching precision ±0.1675 mm considering the average speed of shaking table which was 33.515 mm/s, with a time delay of 0.005 s, ±0.4 mm due to LVDT nonlinearity factor, and ±0.6492 mm from LVDT excitement noise, while the expected photogrammetry monitoring precision is ±0.367 mm which corresponds to a pixel’s pitch in the monitoring frames.

The maximum difference between observations measured using LVDTs system and photogrammetry was found to be 1.251 mm, while the minimum difference was −1.241 mm, resulting in a maximum RE of ±1.251 mm and RMSE of ±0.563 mm, as shown in Figure 14.

**Figure 15:**
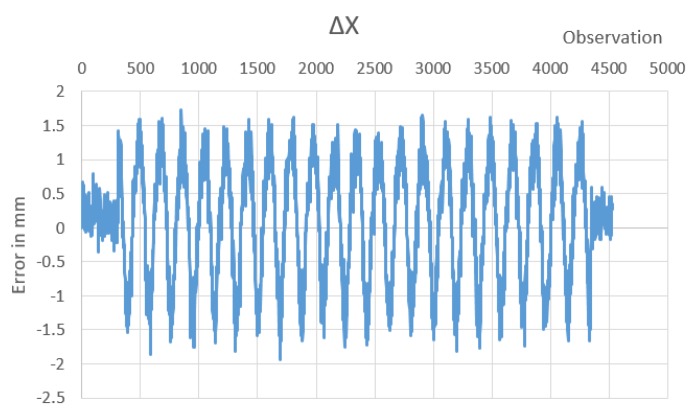
Difference between photogrammetry from the second monitoring station using Faster R-CNN and a Harris corner detector.

The maximum difference between observations measured using photogrammetry applying Harris corner detector and applying Faster R-CNN network was found to be 1.7255 mm, and the minimum observed difference was –1.93743 mm, resulting in maximum RE of ±1.93743 mm, while the measured 4526 observations have RMSE of ±0.868 mm, as shown in Figure 15.

### 3.3. Checking the Reliability of Faster R-CNN Object Detection

**Figure 16:**
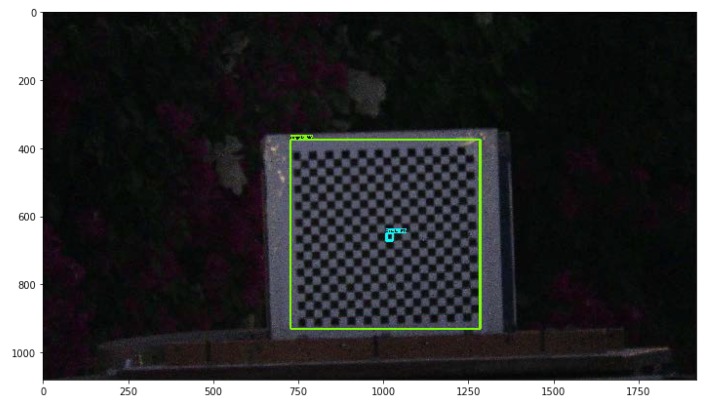
Example of a frame of monitoring video sequence at very low light exposure.

Detecting target points using Harris corner detector proved excellent performance, as shown in previous experiments that were conducted in different environments with a variety of light exposure conditions. However, real-life monitoring conditions can be more challenging, and that is what raised the need for an AI algorithm to help in such scenarios. Faster-R-CNN successfully detected both classes in cases of bright sun and shadow partially covering the target, as shown in Figure A1. To check the reliability of AI for target detection at low light exposure conditions, the test setup presented in Figure 13 was used, while the light source was switched off before the monitoring process. Harris corner detector algorithm could not detect the targets in 3056 out of a monitored 3782 frames. The first successfully detected frame was the fifth frame. On the other hand, the AI aided algorithm detected Target and Track-points in all 3782 monitored frames, as shown in Figure 16.

## 4. Discussion

The precision of monitoring by AI aided algorithm can be improved by implementing a more effective neural network in term of spatial accuracy as convolutional neural networks (CNN), but on the cost of required computational powers and analysis time. As CNN uses a huge number of regions in an input image, resulting in the need for extensive computing powers and limiting the application of CNN on large images, despite consolidating the network layers by max-pooling operations. The same perspective can be applied consonantly using region-based convolutional neural networks (R-CNN) that outcome CNN performance by limiting convolutions to selected 2000 regions [42], or by using the Fast R-CNN algorithm [43]. In conclusion, the Fast R-CNN is considered 25-times faster than R-CNN, while Faster R-CNN is 250-times faster than R-CNN, while a full HD video frame as in Figure 16 consumed about 15 s of analysis using Faster-R-CNN. On the other hand, faster analysis can be achieved using AI aided monitoring with algorithms having higher speed as MobileNet SSD [44] or Yolo [45] algorithms, but at the cost of monitoring spatial accuracy.

## 5. Conclusions

The presented monitoring technique can achieve sub-millimeter precision, with high monitoring rates up to 60 Hz using low-cost non-metric cameras. The implementation of a small to a medium sensor bridge camera, allowed monitoring from apart stable monitoring stations, with adequate precision, depending on the optical zoom capability of the used camera. Moreover, the monitoring system indicates drifts in the target’s local coordinate system, which represents the structure’s local directions. This criterion facilitates the structural health monitoring process. Further analysis testing larger target to camera distances and using various camera models is recommended. 

The AI aided algorithm proved to be reliable in various lighting exposures, and environmental conditions. However, the precision of monitoring by faster R-CNN is lower than that with Harris corner detector, so it is recommended to use AI only in skipped frames, where a Harris corner detector could not detect target points.

The monitoring approach proposed in this research using an AI aided algorithm allows high precision monitoring with a Max RE of less than 2 mm when monitoring from a station about 30 m apart. This system can be considered as a high precision monitoring system compared to commonly used monitoring GPS systems that have standard deviations between 20 and 6 mm [46], and the precision obtained by structural deformations monitoring using close-range photogrammetry [4,47]. Moreover, the introduced monitoring technique precision can be compared to the precision of high-precision sensors in structural monitoring that have RMSE of ±3 mm [48].

## Figures and Tables

**Table 1:** Some of Common Market available bridge cameras [17,18,19].

Model	Canon Powershot SX70HS	Nikon COOLPIX P1000	Sony Cyber-Shot RX10 IV
Sensor	6.17 × 4.55 mm CMOS	6.17 × 4.55 mm CMOS	13.2 × 8.8 mm Stacked BSI
Focal Length (eq. 35 mm)/Aperture	21–1365 mm/f/3.4–6.5	24–3000 mm/f/2.8–8	24–600 mm/f/2.4–4
Optical Zoom	65×	125×	30×
Still Image Resolution	20.3 Megapixels	16 Megapixels	20.1 Megapixels
Still Image bursts	Ten frames per second	Seven frames per second	24 frames per second
Shutter Speed	1/2000–15 s	1/4000–30 s	1/32,000 s
Resolution @ frame rate	3840 × 2160 @ 29.97fps; 1920 × 1080 @ 59.94 fps.	3840 × 2160 @ 30 fps; 1920 × 1080 @ 60 fps.	3820 × 2160 @ 30 fps; 1920 × 1080 @ 120 fps; 1824 × 1026 @ 240 fps.

**Table 2:** Summary of analyzed camera sensors precision for monitoring target 100 m apart.

Camera Model	Canon Powershot SX60HS	Nikon Coolpix P1000
Video monitoring	±1.3 mm in x-dir	±0.596 mm in x-dir
	±1.7 mm in y-dir	±0.782 mm in y-dir
Brust imaging	±0.54 mm in x-dir	±0.248 mm in x-dir
	±0.533 mm in y-dir	±0.244 mm in y-dir

**Table 3:** The summary of network’s evaluation metrics.

Detected Class	Target	Track-Point
Average precision (AP)	100%	96.28%
FP/TP	0/139	2/133
FN/TP	0/139	3/133

**Table 4:** The difference between the proposed monitoring system and LVDT measurements.

Analysis Trail	Analysis 1	Analysis 2
Target tracking	Harris corner detector	Faster-R-CNN (AI)
RMSE	0.32 mm	0.409 mm
RE max	±1.118 mm	±1.535 mm

## Appendix A

The figure shown represents the ability of Faster R-CNN to detect both target and TrackPoint in various light exposures, frame shooting angles, and environments.
